# Coffee Is Associated With Lower Breast Tumor Insulin-Like Growth Factor Receptor 1 Levels in Normal-Weight Patients and Improved Prognosis Following Tamoxifen or Radiotherapy Treatment

**DOI:** 10.3389/fendo.2018.00306

**Published:** 2018-06-06

**Authors:** Sofie Björner, Ann H. Rosendahl, Helga Tryggvadottir, Maria Simonsson, Karin Jirström, Signe Borgquist, Carsten Rose, Christian Ingvar, Helena Jernström

**Affiliations:** ^1^Skåne University Hospital, Department of Clinical Sciences Lund, Oncology and Pathology, Faculty of Medicine, Lund University, Lund, Sweden; ^2^CREATE Health, Department of Immunotechnology, Lund University, Medicon Village, Lund, Sweden; ^3^Skåne University Hospital, Department of Clinical Sciences Lund, Surgery, Lund University, Lund, Sweden

**Keywords:** coffee, insulin-like growth factor receptor 1, body mass index, breast cancer, prognosis

## Abstract

Coffee is associated with decreased breast cancer risk, but the impact of body mass index (BMI) in combination with coffee consumption on prognosis is unclear. The suppressive effect of coffee constituents on the insulin-like growth factor receptor 1 (IGF1R) levels in breast cancer cells may play a role. The aim was to investigate the prognostic impact of coffee consumption and possible associations with tumor-specific IGF1R protein expression and BMI in a population-based cohort in Sweden, comprising 1,014 primary breast cancer patients without pretreatment enrolled 2002–2012 and followed for up to 13 years. Patients with higher coffee consumption had lower tumor IGF1R levels (*P* = 0.025), but only among the normal-weight patients (*P* = 0.005). Coffee did not impact the recurrence-risk overall. However, tamoxifen-treated patients with ER^+^ tumors drinking ≥ 2 cups of coffee/day had lower recurrence-risk [adjusted HR (HR_adj_) 0.57, 95% CI, 0.34–0.97] compared with patients with lower intake, although only among normal-weight patients (HR_adj_ 0.37, 95% CI: 0.17–0.78; *P*_interaction_ = 0.039). Similarly, coffee consumption ≥ 2 cups/day was associated with significantly lower recurrence-risk among the 640 radiotherapy-treated patients irrespective of BMI (HR_adj_ 0.59, 95% CI 0.36–0.98) and in the 296 normal-weight patients (HR_adj_ 0.36, 95% CI 0.17–0.76) but not in the 329 overweight or obese patients (HR_adj_ 0.88, 95% CI 0.42–1.82) although the interaction was not significant (*P*_interaction_ = 0.093). In conclusion, coffee consumption was negatively associated with tumor-specific IGF1R levels only among normal-weight patients. Though, IGF1R did not explain the association between coffee intake and improved prognosis among normal-weight tamoxifen- or radiotherapy-treated patients. Studies of IGF1R-targeting therapies may benefit from taking BMI and coffee consumption into account.

## Introduction

The association between coffee and risk of different types of cancers has been investigated in several studies, and included both lower breast cancer risk and recurrence (1, 2). Results from a meta-analysis concluded that coffee consumption was not associated with overall risk of breast cancer, and that a high coffee consumption may actually decrease the risk of ER^-^ breast cancer (3). A large Swedish study of over 42000 women found that coffee was associated with decreased risk of ER^+^/PgR^−^ breast cancer (4). Coffee consumption may also impact prognosis. We have previously reported that tamoxifen-treated breast cancer patients who drank ≥ 2 cups of coffee/day had a lower recurrence-risk relative to patients with a lower coffee consumption in the present study cohort (5, 6). Potential mechanism for this observation was demonstrated in breast cancer cell lines, where caffeine and caffeic acid suppressed the insulin-like growth receptor 1 (IGF1R) levels and downstream effector mediators. However, no data regarding tumor-specific IGF1R levels in the breast cancer patients were available at the time of that study (6). Tumor-specific IGF1R levels have later been evaluated in this cohort and tumor IGF1R^strong^ protein expression conferred a worse prognosis than weaker expression (7). IGF1R is part of the IGF signaling axis and mediates mitogenic and antiapoptotic signals involving the PI3K-Akt and MAPK-ERK pathways (8). In women, circulating IGF-1 levels appear to be negatively associated with coffee consumption (9). In addition to the growth promoting effects, the IGF axis is further involved in metabolic processes and a positive association between increased body mass index (BMI) and IGF1R mRNA expression in postmenopausal breast cancer tissue has been reported (10). Taken together, both BMI and coffee consumption may be of importance in breast cancer. A previous study suggested that coffee might be associated with a lower risk for endometrial cancer, but only among obese postmenopausal women (11). In the light of the ongoing global obesity epidemic, there is a need to better understand the interplay between daily diet and physical conditions in order to possibly prevent breast cancer recurrence. Whether the association between coffee intake and recurrence-risk is modified by BMI and tumor-specific IGF1R expression in breast cancer patients has to our knowledge not been studied. IGF1R targeting treatments have been disappointing in the clinical setting, and it has been suggested that pathophysiology related to obesity should be taken into account (12, 13).

We hypothesize that coffee may play a role for breast cancer prognosis through modulation of tumor IGF1R protein expression in human breast cancer, and that the impact of coffee may be modified by BMI given that both coffee and BMI influence circulating IGF-1 levels in women. The aim of this study was to evaluate if the prognostic impact of coffee consumption overall, and especially in tamoxifen-treated breast cancer patients, was partly mediated through associations with tumor-specific IGF1R protein expression and the patients’ BMI.

## Materials and Methods

### Patients

The 1,116 patients in the present study are part of an ongoing population-based cohort, BC Blood Study, and were included between October 2002 and June 2012 at the Skåne University Hospital, Lund, Sweden (5). The patients, 24–99 years old, were diagnosed with primary breast cancer without other history of cancer within the last 10 years. Body measurements were obtained by a research nurse prior to surgery. Patients were also asked to fill out preoperative questionnaires regarding medications, reproductive history, and life style factors such as smoking and coffee consumption. Information regarding clinicopathological factors including hormone receptor status, tumor size, lymph node status, histologic grade were obtained from pathology reports and medical records. Information on breast cancer recurrence or death was collected from pathology reports, patient charts, the Population Registry, or the Regional Tumor Registry. Patients may have received more than one type of adjuvant treatment during follow-up. Adjuvant breast cancer treatment was administered according to standard of care and was recorded until the first breast cancer event. In patients without any breast cancer events, treatments were recorded until last follow-up or death prior to July 1, 2016. This study was carried out in accordance with the recommendations of the local ethics committee at Lund University with written informed consent from all patients. The study was approved by the local ethics committee at Lund University (Dnr 75-02, Dnr 37-08, Dnr 658-09, Dnr 58-12, Dnr 379-12, Dnr 227-13, Dnr 277-15, and Dnr 458-15).

**Figure 1:**
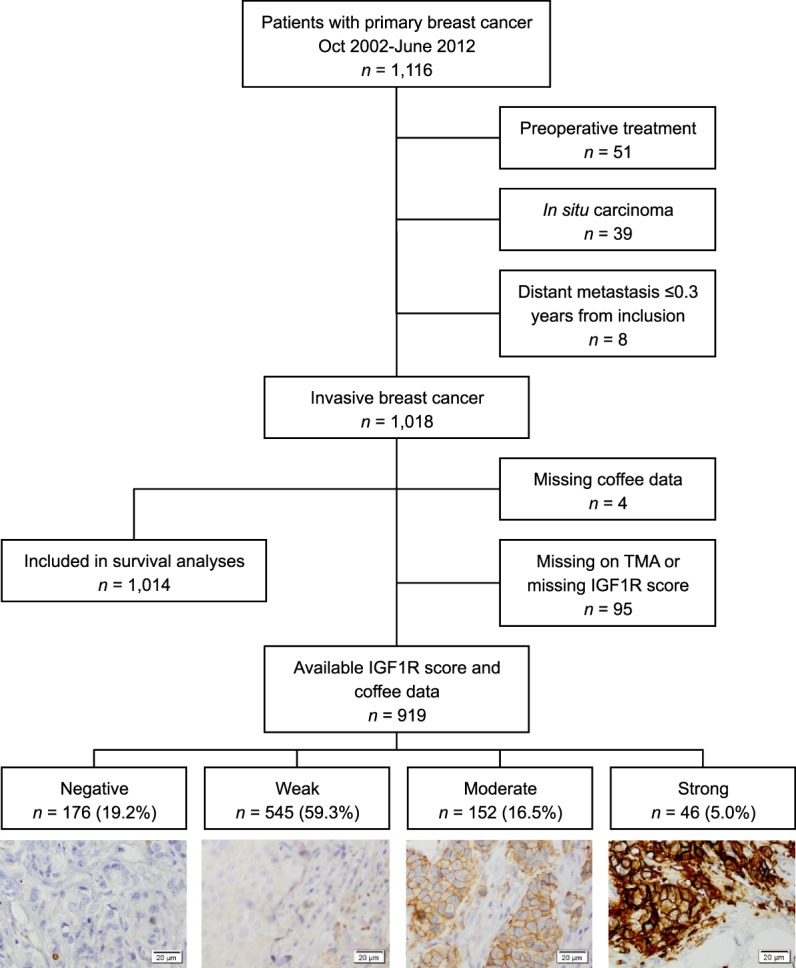
Flowchart of included and excluded patients and different levels of insulin-like growth factor receptor 1 staining intensities.

The final study cohort consisted of 1,014 patients after excluding patients who received preoperative treatment (*n* = 51), patients with only ductal carcinoma *in situ* (*n* = 39), patients with distant metastasis ≤ 0.3 years from inclusion (*n* = 8), and patients without data on coffee consumption (*n* = 4). For analyses involving tumor-specific IGF1R expression, tumors from 95 patients were missing on the tissue microarray (TMA) or had missing IGF1R score (Figure 1.). The report followed the Reporting recommendations for tumor MARKer prognostic studies (REMARK) criteria (14).

### Immunohistochemistry

Immunohistochemical staining for IGF1R has been described previously (7). Briefly, TMA containing dual 1.0 mm cores from representative tumor regions from surgical specimens of formalin-fixed paraffin-embedded tissue blocks were constructed by a semi-automated tissue array device (Beeches instruments, Sun Prairie, WI, USA). The 4 µm thick sections were automatically deparaffinized and pretreated using the PT Link system (DAKO, Glostrup, Denmark). The sections were stained with anti-IGF1Rβ antibody (sc-713, Santa Cruz Biotechnology; dilution 1:150) using an Autostainer Plus from DAKO with EnVision FLEX high-pH kit according to the manufacturer’s instructions (DAKO, Glostrup, Denmark). Scoring was performed by two independent observers (Sofie Björner and Ann H. Rosendahl) and re-examination was performed in case of discrepancy (7.2%) until consensus was reached. A combined four level score of cytoplasmic and membrane staining intensity was used; negative, weak, moderate, strong. The highest score was applied in case of bilateral tumors (*n* = 15) and all scores came from the same tumor.

### Statistical Analysis

All statistical analyses were performed using the SPSS software version 22 (IBM). The linear-by-linear association test was used for analyses between three levels of coffee consumption, low (0–1 cup/day), moderate (2–4 cups/day), and high (≥5 cups/day), and categorical variables. The Jonckheere-Terpstra test was used for analyses between coffee consumption and continuous variables including the potential association between time between surgery and staining (years) and combined cytoplasmic and membrane staining intensity of IGF1R. There was a significant association between longer time since surgery and weaker IGF1R expression (*P* < 0.001) and time since surgery was, therefore, added as an adjustment variable for analyses of IGF1R and other parameters. Partial correlation test with adjustment for time between surgery and IGF1R staining was used for association analyses between IGF1R expression and coffee consumption.

In the survival analyses, Kaplan–Meier curves and LogRank tests were used for univariable analyses and Cox regression was used for multivariable analyses providing hazard ratios with 95% confidence intervals (CI) adjusted for age (continuous), invasive tumor size (≥21 mm or skin or muscular involvement), any axillary lymph node involvement, histological grade III, ER status, and smoking. Two-way interaction terms between coffee consumption and BMI (≥25 kg/m^2^) as well as coffee consumption and ER status were calculated and used in adjusted Cox regression analyses to investigate potential effect modifications.

Patients were followed from inclusion to the first breast cancer event, distant metastasis or death, respectively. Breast cancer events were defined as local or regional recurrences, contralateral cancer, or distant metastasis. The time to event is referred to as breast cancer-free interval (BCFI). Patients without breast cancer events were censored at the last follow-up or death prior to July 1st, 2016.

## Results

### Coffee Consumption and Associations With Tumor and Patient Characteristics

Associations between coffee consumption and tumor and patient characteristics among all patients are presented in Table 1, as well as for patients stratified by BMI (cutoff ≥ 25 kg/m^2^) in Table 2. Coffee consumption was categorized into low (0–1 cup/day), moderate (2–4 cups/day), and high (≥5 cups/day). Patients with higher coffee consumption had significantly lower tumor IGF1R levels compared with patients with lower coffee consumption (*P* = 0.025). This was, however, only observed among the normal-weight patients (*P* = 0.005) after stratification according to BMI. As previously reported (6), patients with higher coffee consumption had smaller tumors (*P*_trend_ = 0.038), although this was limited to overweight or obese patients (*P*_trend_ = 0.005). There was a borderline negative trend between coffee intake and ER positivity. There were significant positive trends between smoking and coffee consumption (*P*_trend_ < 0.001) both among normal-weight (*P*_trend_ < 0.001) and overweight or obese patients (*P*_trend_ < 0.001). Among overweight or obese patients, HER2 status was associated with coffee consumption (*P*_trend_ = 0.040). Age, BMI, and smoking were not associated with IGF1R levels in the present cohort (all *P*-values > 0.25).

**Table 1:** Tumor and patient characteristics of 1.014 patients in relation to daily coffee consumption (cups/day).

	All (*n* = 1,014) *n* (%)	Missing (*n*)	Low (0–1) (*n* = 184) *n* (%)	Moderate (2–4) (*n* = 602) *n* (%)	High (5+) (*n* = 200) *n* (%)	*P*_trend_
**Tumor characteristics, *n* (%)**						
Insulin-like growth factor receptor 1		95				
Negative	176 (19.2)		24 (13.6)	111 (20.0)	41 (21.7)	0.025^a^
Weak	545 (59.3)		104 (59.1)	332 (59.9)	109 (57.7)	
Moderate	152 (16.5)		35 (19.9)	86 (15.5)	31 (16.4)	
Strong	46 (5.0)		13 (7.4)	25 (4.5)	8 (4.2)	
Hormone receptor status						
ER^+^	891 (88.0)	1	173 (91.1)	542 (87.8)	176 (85.4)	0.087^b^
PR^+^	720 (71.1)	1	130 (68.4)	446 (72.3)	144 (69.9)	>0.3^b^
Invasive tumor size		0				
≤20 mm	735 (72.5)		125 (65.8)	455 (73.6)	155 (75.2)	0.038^b^
≥21 mm and skin or muscular involvement independent of size	279 (27.5)		65 (34.2)	163 (26.4)	51 (24.8)	
Lymph node status		2				
0	624 (61.7)		107 (56.6)	390 (63.2)	127 (61.7)	0.25^b^
1–3	302 (29.8)		62 (32.8)	177 (28.7)	63 (30.6)	
4+	86 (8.5)		20 (10.6)	50 (8.1)	16 (7.8)	
Histologic grade		1				
I	254 (25.1)		40 (21.1)	159 (25.8)	55 (26.7)	>0.3^b^
II	507 (50.0)		105 (55.3)	302 (48.9)	100 (48.5)	
III	252 (24.9)		45 (23.7)	156 (25.3)	51 (24.8)	
HER2 status^c^		49				
Positive	79 (12.1)		10 (8.0)	48 (12.5)	21 (14.6)	0.10^b^
**Patient characteristics, median (IQR)**						
Age	61.1 (52.1–68.1)		59.1 (48.5–67.9)	62.6 (55.0–69.3)	58.6 (49.4–65.4)	0.30^d^
Body mass index (kg/m^2^)	25.1 (22.5–28.3)	28	24.4 (21.9–27.7)	25.2 (22.7–28.6)	25.1 (22.5–28.1)	0.30^d^
Current smoker, *n* (%)	204 (20.2)	2	17 (8.9)	111 (18.0)	76 (36.9)	<0.001^b^

*^a^Partial correlation test adjusted for time since surgery*.

*^b^Linear-by-linear association test*.

*^c^HER2 status was only available for patients included as of November 2005*.

*^d^Jonckheere-Terpstra test*.

*IQR, interquartile range*.

**Table 2:** Selected tumor and patient characteristics of 986 patients stratified by body mass index (BMI) in relation to daily coffee consumption (cups/day).

	All (*n* = 986) *n*(%)	Missing (*n*)	Low (0–1) (*n* = 184) *n*(%)	Moderate (2–4) (*n* = 602) *n*(%)	High (5+) (*n* = 200) *n*(%)	*P*_trend_
BMI < 25 kg/m^2^ (*n* = 487)			(*n* = 98)	(*n* = 291)	(*n* = 98)	
Insulin-like growth factor receptor 1 (IGF1R)		51				
Negative	96 (22.0)		13 (14.6)	59 (22.8)	24 (27.3)	0.005^a^
Weak	252 (57.8)		48 (53.9)	152 (58.7)	52 (59.1)	
Moderate	65 (14.9)		22 (24.7)	34 (13.1)	9 (10.2)	
Strong	23 (5.3)		6 (6.7)	14 (5.4)	3 (3.4)	
Hormone receptor status		0				
ER^+^	440 (90.3)		89 (90.8)	263 (90.4)	88 (89.8)	>0.3^b^
Invasive tumor size		0				
≤20 mm	385 (79.1)		76 (77.6)	230 (79.0)	79 (80.6)	>0.3^b^
≥21 mm and skin or muscular involvement independent of size	102 (20.9)		22 (22.4)	61 (21.0)	19 (19.4)	
Lymph node status		2				
0	315 (64.9)		58 (59.8)	193 (66.6)	64 (65.3)	>0.3^b^
1–3	138 (28.5)		30 (30.9)	81 (27.9)	27 (27.6)	
4+	32 (6.6)		9 (9.3)	16 (5.5)	7 (7.1)	
Histologic grade		1				
I	137 (28.2)		25 (25.5)	81 (27.9)	31 (31.6)	>0.3^b^
II	242 (49.8)		50 (51.0)	145 (50.0)	47 (48.0)	
III	107 (22.0)		23 (23.5)	64 (22.1)	20 (20.4)	
HER2 status						
Positive	39 (12.8)	25	7 (10.8)	24 (13.7)	8 (12.5)	>0.3^b^
Age (median, IQR)	59.5 (50.6–67.2)		58.5 (48.1–67.9)	60.8 (52.7–68.1)	55.6 (47.5–64.6)	0.19^c^
Current smoker	108 (22.2)	1	7 (7.1)	67 (23.1)	34 (34.7)	<0.001^b^

BMI ≥ 25 kg/m^2^ (*n* = 499)			(*n* = 86)	(*n* = 311)	(*n* = 102)	
IGF1R		42				
Negative	79 (17.3)		11 (13.4)	51 (18.2)	17 (17.9)	>0.3^a^
Weak	277 (60.6)		52 (63.4)	170 (60.7)	55 (57.9)	
Moderate	80 (17.5)		13 (15.9)	48 (17.1)	19 (20.0)	
Strong	21 (4.6)		6 (7.3)	11 (3.9)	4 (4.2)	
Hormone receptor status		1				
ER^+^	426 (85.5)		78 (90.7)	263 (84.8)	85 (83.3)	0.17^b^
Invasive tumor size		0				
≤20 mm	331 (66.3)		44 (51.2)	214 (68.8)	73 (71.6)	0.005^b^
≥21 mm and skin or muscular involvement independent of size	168 (33.7)		42 (48.8)	97 (31.2)	29 (28.4)	
Lymph node status		0				
0	289 (57.9)		43 (50.0)	187 (60.1)	59 (57.8)	0.26^b^
1–3	157 (31.5)		32 (37.2)	91 (29.3)	34 (33.3)	
4+	53 (10.6)		11 (12.8)	33 (10.6)	9 (8.8)	
Histologic grade		0				
I	112 (22.4)		13 (15.1)	76 (24.4)	23 (22.5)	>0.3^b^
II	250 (50.1)		51 (59.3)	148 (47.6)	51 (50.0)	
III	137 (27.5)		22 (25.6)	87 (28.0)	28 (27.5)	
HER2 status^c^		24				
Positive	40 (11.5)		3 (5.0)	24 (11.5)	13 (16.3)	0.040^b^
Age (median, IQR)	62.4 (54.1–69.4)		59.7 (48.7–67.7)	63.7 (57.6–70.4)	59.9 (51.9–66.6)	>0.3^d^
Current smoker	90 (18.1)	1	9 (10.5)	42 (13.5)	39 (38.2)	<0.001^b^

*Information about BMI was missing for 28 patients*.

*^a^Partial correlation test adjusted for time between surgery and staining*.

*^b^Linear-by-linear association test*.

*^c^HER2 status was only available for patients included as of November 2005*.

*^d^Jonckheere-Terpstra test*.

*IQR, interquartile range*.

### Coffee Consumption and Prognosis in Relation to BMI and Tumor IGF1R

The prognostic impact of coffee consumption was studied in a breast cancer cohort where patients were followed for up to 13 years, with a median follow-up of 5.4 years for patients still at risk. Of the 1,014 patients, 155 patients had been diagnosed with a first breast cancer event (local, regional, contralateral, or distant), and of these patients, 98 had a distant metastasis. Of the 127 patients who died during the study period, 74 patients had a breast cancer event prior to death.

**Figure 2:**
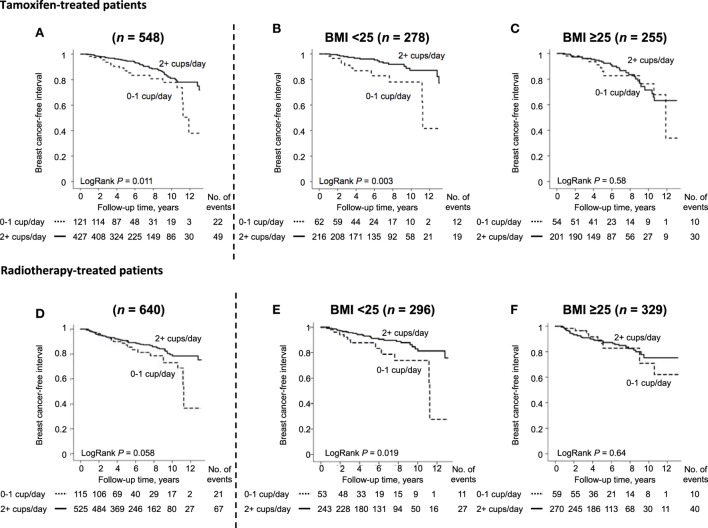
Breast cancer-free interval in tamoxifen-treated patients with ER+ tumors, **(A)** irrespective of body mass index (BMI), **(B)** in patients with BMI < 25 kg/m^2^, **(C)** in patients with BMI ≥ 25 kg/m^2^ and in radiotherapy-treated patients irrespective of ER status, **(D)** irrespective of BMI, **(E)** in patients with BMI < 25 kg/m^2^, **(F)** in patients with BMI ≥ 25 kg/m^2^. As this is an ongoing study, there are fewer patients with longer follow-up times.

This study confirmed the previous findings that coffee did not impact the overall recurrence-risk (LogRank *P* = 0.22). There was no significant effect modification of coffee consumption on recurrence-risk depending on tumor ER status. However, drinking ≥ 2 cups of coffee/day was associated with a favorable prognosis among tamoxifen-treated patients with ER^+^ tumors (Figure 2A), but was not significantly associated with prognosis in AI- (LogRank *P* = 0.48), chemo- (LogRank *P* = 0.85), and/or radiotherapy-treated patients (LogRank *P* = 0.058) in the univariable models. Multivariable analysis demonstrated that coffee consumption was an independent recurrence-risk predictor for tamoxifen-treated patients with ER^+^ tumors after adjustment for age, clinicopathological factors, and smoking [adjusted HR (HR_adj_) 0.57, 95% CI, 0.34–0.97]. Additionally, among tamoxifen-treated patients, there was a significant effect modification depending on BMI on the associations between coffee intake and BCFI (*P*_interaction_ = 0.039). After stratification by BMI status, ≥2 cups of coffee/day remained associated with lower recurrence-risk among the 278 normal-weight patients (HR_adj_ 0.37, 95% CI: 0.17–0.78; Figure 2B), but not among the 255 overweight or obese patients (HR_adj_ 0.94, 95% CI: 0.44–2.03; Figure 2C). Though, multivariable analysis suggested that the reduced recurrence-risk among normal-weight tamoxifen-treated patients with ER^+^ tumors with known IGF1R tumor expression (*n* = 254) could not be explained by decreased IGF1R tumor expression (adjusted *P*_trend_ = 0.97). This was also true when using a cut-off of IGF1R^strong^ (*n* = 16) versus weaker protein expression (adjusted *P* = 0.18). Results from the multivariable analyses in relation to four groups of IGF1R protein expression are presented in Table 3.

**Table 3:** Multivariable analysis of risk of breast cancer events in tamoxifen-treated patients with ER^+^ tumors (*n* = 548).

	All patients			Body mass index (BMI) < 25 kg/m^2^			BMI ≥ 25 kg/m^2^		
		
	HR	95% CI		HR	95% CI		HR	95% CI	
		Lower	Upper		Lower	Upper		Lower	Upper
Age	0.98	0.96	1.01	0.99	0.95	1.03	0.98	0.94	1.01
Invasive tumor size ≥ 21 mm or muscular/skin involvement	2.38	1.42	3.97	1.87	0.78	4.50	2.32	1.16	4.65
Any axillary nodal involvement	1.04	0.62	1.73	0.96	0.44	2.09	1.05	0.52	2.13
Histological grade III	1.33	0.73	2.41	1.42	0.54	3.71	1.43	0.62	3.30
Current smoking	1.03	0.53	2.01	0.54	0.16	1.85	1.69	0.72	3.93
Coffee consumption ≥ 2 cups/day	0.58	0.34	0.99	0.37	0.17	0.82	0.93	0.43	2.04
Insulin-like growth factor receptor 1 protein expression	0.96	0.70	1.32	0.99	0.59	1.66	0.96	0.63	1.48

*In all patients, 47 missed data on one or more variables. In patients with BMI < 25 kg/m^2^, 25 missed data on one or more variables. In patients with BMI ≥ 25 kg/m^2^, 21 missed data on one or more variables*.

Since there was a borderline significant association between coffee intake and recurrence-risk in radiotherapy-treated patients in the univariable model, a multivariable analysis was also performed. Coffee consumption ≥ 2 cups/day was associated with significantly lower recurrence-risk among all 640 patients (HR_adj_ 0.59, 95% CI 0.36–0.98; Figure 2D) and in the 296 normal-weight patients (HR_adj_ 0.36, 95% CI 0.17–0.76; Figure 2E) but not in the 329 overweight or obese patients (HR_adj_ 0.88, 95% CI 0.42–1.82; Figure 2F) although the interaction was not significant (*P*_interaction_ = 0.093). Again, the lower recurrence-risk in normal-weight radiotherapy-treated patients could not be explained by IGF1R expression in the multivariable model (*P*_trend_ = 0.27) as shown in Table 4.

**Table 4:** Multivariable analysis of risk of breast cancer events in radiotherapy-treated patients irrespective of tumor ER status (n = 640).

	All patients			Body mass index (BMI) < 25 kg/m^2^			BMI ≥ 25 kg/m^2^		
		
	HR	95% CI		HR	95% CI		HR	95% CI	
		Lower	Upper		Lower	Upper		Lower	Upper
Age	0.99	0.97	1.01	0.97	0.94	1,00	1,00	0.97	1.03
Invasive tumor size ≥ 21 mm or muscular/skin involvement	2.12	1.31	3.42	2.16	0.94	4.92	2.26	1.20	4.28
Any axillary nodal involvement	1.30	0.82	2.08	1.20	0.60	2.42	1.51	0.80	2.85
Histological grade III	1.63	1.02	2.58	1.29	0.61	2.71	1.69	0.92	3.11
Current smoking	1.91	1.17	3.14	1.86	0.88	3.91	2.17	1.10	4.27
Coffee consumption ≥ 2 cups/day	0.57	0.34	0.95	0.36	0.17	0.76	0.81	0.38	1.70
Insulin-like growth factor receptor 1 protein expression	0.89	0.67	1.19	0.78	0.50	1.21	0.98	0.66	1.46

*In all patients, 50 missed data on one or more variables. In patients with BMI < 25 kg/m^2^, 25 missed data on one or more variables. In patients with BMI ≥ 25 kg/m^2^, 23 missed data on one or more variables*.

## Discussion

The present study suggests that patients’ BMI in relation to coffee intake plays a preventive role for breast cancer recurrence among tamoxifen-treated primary breast cancer patients with ER + tumors or radiotherapy treated patients irrespective of ER status. The study further presents a significant negative correlation between coffee consumption and IGF1R levels in primary breast cancer among normal-weight, but not overweight or obese patients. However, the IGF1R level did not as an individual factor explain the association between coffee intake and clinical outcome.

Coffee has previously been associated with both lower breast cancer risk and recurrence (1, 2), especially among tamoxifen-treated breast cancer patients with ER^+^ tumors (5,6). The impact of coffee consumption on breast cancer prognosis in this patient cohort has previously been reported, but with shorter follow-up times and different inclusion periods (5, 6). The number of tamoxifen-treated patients was larger in the present study due to the longer follow-up. Furthermore, the present study demonstrated that it was only in the normal-weight tamoxifen-treated patients with ER^+^ tumors that coffee was associated with decreased recurrence-risk. No significant prognostic impact of coffee consumption was observed among overweight or obese patients. Why coffee consumption was associated with lower tumor-specific IGF1R levels only among the normal-weight patients stands in contrast to the results found in endometrial cancer patients, where coffee was beneficial in the heavier patients (11). Tamoxifen and coffee appear to be associated with diabetes and the insulin pathway, although in opposite directions. Tamoxifen, but not aromatase inhibitors, increases the risk for diabetes (15) and an *in vivo* study suggests that tamoxifen is associated with lower total IGF1R levels but higher phosphorylated IGF1R levels (16). While higher BMI is associated with increased proinsulin, insulin, and C-peptide in women (17), caffeinated coffee lowered C-peptide more in overweight and obese women compared to normal-weight women (18) and reduced the risk of diabetes type 2 (2). Further, a higher BMI was also associated with higher estradiol, particularly in tumor tissue relative to serum levels, in patients with ER^+^ tumors, but not ER^−^ tumors (19). It is possible that overweight and obese patients have altered intracellular receptor trafficking and recycling rates depending on ligand levels (20) that may have impacted the results such that coffee only affected IGF1R levels in normal-weight patients. We did not have access to tumor tissue after tamoxifen exposure and could, therefore, not examine whether there was a differential tamoxifen response with respect to ER and IGF1R levels between normal-weight patients and those with higher BMI. The current study is to our knowledge the first study among breast cancer patients that investigates the importance of taking BMI into account when evaluating the prognostic impact of coffee consumption.

In line with previously demonstrated biological mechanisms by coffee constituents in suppressing IGF1R levels in breast cancer cells *in vitro* (6), the present study further revealed that increasing coffee consumption was associated with lower tumor-specific IGF1R levels also in primary breast cancer. However, this observation appeared limited to normal-weight patients. Multivariable models, showed no significant impact of IGF1R on breast cancer outcome, suggesting that lower IGF1R levels could not independently explain the relationship between coffee consumption and clinical outcome in tamoxifen-treated patients. In addition to suppressing IGF1R levels in breast cancer cells, caffeine reduced the key downstream effector pAkt levels as well as the growth promoting ER and cyclin D1 abundance in ER^+^ cells *in vitro* (6). Furthermore, coffee also influenced the levels of enzymes associated with tamoxifen activation (21). The impact of coffee on prognosis appeared to be limited to tamoxifen-treated patients with ER^+^ tumors or radiotherapy-treated patients, since the association was not observed among patients treated with AI and/or chemotherapy. The crosstalk between the IGF and ER signaling pathways (22, 23) have resulted in treatment strategies targeting both the IGF signaling axis and ER (24). The observed impact of coffee consumption on tumor-specific IGF1R levels in relation to BMI suggests that coffee intake as well as BMI could be of importance in studies regarding IGF1R targeting treatments.

The association between coffee intake and ER status has been investigated in studies where the preventive beneficial effect of coffee has both been more pronounced among patients with ER^+^ tumors (4) and in patients with ER^-^ tumors (3). In the present study, there was no significant effect modification depending on ER status. Exploratory analyses in patients stratified by ER status resulted in no difference in prognostic impact of coffee between the groups (data not shown). The present study did only detect a borderline significant negative trend between the amount of coffee intake and ER status compared to the previous studies based on different number of patients from the same cohort (5, 6). This may be due to variations in other factors that also impact ER status, such as age and smoking that are linked to both coffee intake and ER status (25, 26).

In conclusion, a higher coffee consumption was associated with lower tumor-specific IGF1R expression, although only among normal-weight patients. The observed lower recurrence-risk among tamoxifen-treated patients with ER^+^ tumors or radiotherapy-treated patients with moderate to high coffee intake was also restricted to normal-weight patients. However, the lower IGF1R levels did not independently explain the favorable impact of coffee consumption in combination with tamoxifen or radiotherapy on clinical outcome. Since coffee and BMI both impact circulating IGF-1 levels, these factors may need to be taken into account when evaluating IGF1R targeting treatments.

## Ethics Statement

This study was carried out in accordance with the recommendations of the ethics committee at Lund University. All subjects gave written informed consent in accordance with the Declaration of Helsinki. The study was approved by the ethics committee at Lund University (Dnr 75-02, Dnr 37-08, Dnr 658-09, Dnr 58-12, Dnr 379-12, Dnr 227-13, Dnr 277-15, and Dnr 458-15).

## Author Contributions

Conception and Design: SBj, AR, CR, CI, and HJ. Development of methodology and analysis and interpretation of data: SBj, AR, and HJ. Acquisition of data: SBj, AR, HT, MS, CI, and HJ. Administrative, technical, or material support: SBj, AR, HT, MS, KJ, and HJ. Study supervision: CI and HJ. Writing, review, and/or revision of the manuscript: SBj, AR, HT, MS, KJ, SB, CR, CI, and HJ.

## Conflict of Interest Statement

The authors declare that the research was conducted in the absence of any commercial or financial relationships that could be construed as a potential conflict of interest.
